# Ocular findings in Noonan syndrome: a retrospective cohort study of 105 patients

**DOI:** 10.1007/s00431-018-3183-1

**Published:** 2018-06-09

**Authors:** Dorothée C. van Trier, Ineke van der Burgt, Renske W. Draaijer, Johannes R. M. Cruysberg, Cees Noordam, Jos M. Draaisma

**Affiliations:** 1 Department of Pediatrics, Radboud University Medical Center Amalia Children’s hospital, P.O. Box, 9101, 6500 HB Nijmegen, The Netherlands; 2 Department of Human Genetics, Radboud University Medical Center, Nijmegen, The Netherlands; 3 Department of Ophthalmology, Radboud University Medical Center, Nijmegen, The Netherlands

**Keywords:** Noonan syndrome, Genotype-phenotype correlation, Ocular abnormalities, Refractive error, Visual impairment, Optic nerve abnormalities, Optic atrophy, Optic nerve hypoplasia, Nystagmus

## Abstract

The aim of this retrospective study is to describe ocular findings in a large Noonan syndrome cohort and to detect associations between ocular features and genetic mutations that were not found in earlier studies. We collected ophthalmological and genetic data of 105 patients (median age, 12 years; range, 0–60 years) clinically diagnosed as Noonan syndrome. The ocular findings were linked to the genotypes. All patients with Noonan syndrome showed multiple abnormalities in the categories of vision and refraction, external ocular features, ocular alignment and motility, anterior ocular segment, and posterior ocular segment. In total, 50 patients have NS due to a mutation in *PTPN11*. Permanent visual impairment (bilateral best-corrected visual acuity < 0.3) was found in 7 patients, including patients with a mutation in *RAF1*, *SHOC2*, and *KRAS*. Keratoconus was found in 2 *PTPN11* positive patients, and prominent corneal nerves were observed in a patient with a *SOS1* mutation.

*Conclusions*: This study shows an overview of ocular abnormalities in Noonan syndrome, including permanent visual impairment caused by binocular optic nerve abnormalities and nystagmus. Delay in ophthalmological diagnosis is still present, also in patients with visual impairment. All Noonan syndrome patients should have a complete ophthalmological examination at the time of diagnosis.

**Table:** 

**What is Known:** *• Although we discover more pathogenic mutations in patients with Noonan syndrome, Noonan syndrome still is a clinical diagnosis* *• Ocular features of Noonan syndrome are characterized by developmental anomalies of the eyelids and associated with other ocular abnormalities in childhood (including refractive errors, strabismus and amblyopia).*	
**What is New:** *• There seems to be a delay in the ophthalmological diagnosis and awareness of the broad variety ofophthalmological features including refractive errors and visual impairment in Noonan syndrome is needed. All children should have a full ophthalmological examination at the time of diagnosis.* *• Permanent visual impairment (best-corrected visual acuity < 0.3) is found in patients with mutations in RAF1, SHOC2, and KRAS and the cause is probably a developmental disorder of the optic nerves.*

## Introduction

Noonan syndrome (NS) is an autosomal dominant disorder with a great variety in phenotype. Characteristics described with high frequency include short stature, congenital heart defects, face dysmorphology, and mild developmental delay. Other manifestations are cryptorchidism in males, chest wall abnormalities and ophthalmological abnormalities [19]. NS belongs to the Rasopathies. Different mutations in coding genes leading to dysregulation of the Ras/mitogen-activated protein kinase pathway can cause NS. The first gene discovered was *PTPN11* on chromosome 12q24.1 [17]. Gain-of-function mutations in *PTPN11* are found in approximately 50% of the NS patients [18]. At the moment, more than 14 genes responsible for NS are elucidated, most frequent *PTPN11* (50%), *SOS1* (10–13%), and other less frequent genes including *KRAS*, *RAF1*, and *RIT1* [4, 11, 14, 16]. Facial dysmorphology is one of the major used clinical criteria for NS [19] and external ocular abnormalities (including hypertelorism, epicanthic folds, ptosis, and downslanting palpebral fissures) play an important role in facial characteristics. Besides the external ocular manifestations, other ocular manifestations occur and the results of ophthalmic examinations in NS are described briefly. The first cohort is described in 1992 with ophthalmological examinations in 58 patients [8]. Few other studies report on ocular examinations [1, 10, 12] and the most recent study is a prospective ocular examination performed in our tertiary referral center in 25 patients that shows at least three ocular features in more than 95% of NS patients [20]. In the international NS clinical management guidelines, referral to an ophthalmologist for assessment at the point of diagnosis is recommended [9, 13, 15]. To give a more extensive overview of ophthalmological abnormalities and ocular problems in NS patients, we collected ophthalmological data in a large retrospective cohort.

## Materials and methods

We collected retrospective data in a NS population at the Radboud University Medical Center in the Netherlands. All patients were clinically diagnosed, fulfilling the Van der Burgt criteria [19]. In total, 201 patients were asked for informed consent and 128 responded and gave permission for the use of their data. All available ophthalmological, pediatric, and genetic data of the participating patients were collected and used. Children were defined as younger than 18 years old. Patients without available data or who never visited an ophthalmologist/pediatrician were excluded.

The primary outcome of this study was the presence of ocular abnormalities in a NS population and ophthalmological, pediatric, and genetic data were used. The ocular outcomes were linked to the genotypes. In the patients without a known mutation, there were either no genetic analyses done or (until now) no mutations identified after testing (a selection of) the NS genes. Since NS is a clinical diagnosis, and in 15–20% of the patients no causative mutation is known, we included all patients in our study. We included all registered ocular abnormalities in the categories vision and refraction, external ocular features, ocular alignment and motility, anterior ocular segment, and posterior ocular segment. No statistical analyses were used and the results were descriptive.

## Results

There were 128 patients who gave permission for the use of their data. In 14 patients, no ophthalmological data were available and 9 of 128 patients (7%) were excluded because they never visited an ophthalmologist. A total of 105 patients were included and 68 of them were younger than 18 years old. The median age of the cohort was 12 years, with a range between 1 and 60 years. Genetic examinations showed a causative mutation for NS in 78 patients.

The ocular features and gene mutations of the 105 NS patients are shown in Table 1. Seven patients were visually impaired, defined as binocular best-corrected visual acuity (BCVA) lower than 0.3, mainly attributable to binocular optic nerve abnormalities and manifest nystagmus These patients had a mutation in the *RAF1* gene (1 patient), *SHOC2* gene (2 patients), or *KRAS* gene (2 patients); in 1 patient, no mutation was identified after genetic testing and another patient had no genetic analysis. These 7 patients are shown comprehensively in Table 2. Amblyopia, defined as visual loss caused by visual deprivation (e.g., by strabismus, refractive errors, cataract, or ptosis) in childhood, was reported in 28 patients.

**Table 1:** Ocular features of mutation-positive and mutation-negative patients with Noonan syndrome

(retrospective study; *n* = 105)									
	Total	*PTPN11*	*SOS1*	*RAF1*	*SHOC2*	*MAP2K2*	*KRAS*	*A2ML*	No mutation
	*n* = 105	*n* = 50	*n* = 10	*n* = 7	*n* = 5	*n* = 1	*n* = 4	*n* = 1	*n* = 27
Vision and refraction									
Visually impaired (both eyes BCVA < 0.3)	7	0	0	1	2	0	2	0	2
Amblyopia (including in history)	28	12	2	3	0	1	2	0	8
Myopia (SEA ≥ 1D)	25	10	4	2	2	0	2	1	4
High myopia (≥ 5D)	5	3	0	0	0	0	1	0	1
Hyperopia (SEA ≥ 1D)	39	23	1	1	2	1	2	0	9
High hyperopia (≥ 5D)	2	1	0	0	0	0	0	0	1
Astigmatism (≥ 1D)	35	21	1	3	0	1	1	0	8
High astigmatism (≥ 5D)	3	2	0	0	0	0	0	0	1
External ocular features									
Hypertelorism	63	30	8	6	2	1	1	1	14
Ptosis	55	21	8	5	1	1	2	1	16
Epicanthic folds	32	13	3	2	1	1	1	0	11
Downslanting palpebral fissures	39	18	5	5	1	1	0	0	9
Upslanting palpebral fissures	2	1	0	0	0	0	0	0	1
Proptosis/pseudo-proptosis	8	3	1	1	1	0	0	0	2
Strabismus and ocular motility									
Strabismus	40	17	0	3	4	1	4	0	11
Limited ocular motility	9	2	0	2	2	1	0	0	2
Nystagmus	16	5	1	3	2	0	3	0	2
Anterior ocular segment									
Keratoconus	4	2	0	0	0	0	0	0	2
Prominent corneal nerves	2	0	1	0	0	0	0	0	1
Corneal opacities	1	0	0	0	0	0	0	1	0
Posterior embryotoxon	2	1	0	1	0	0	0	0	0
Iris coloboma	1	1	0	0	0	0	0	0	0
Cataract	3	1	1	1	0	0	0	0	0
Posterior ocular segment									
Optic nerve head abnormalities	10	5	1	0	1	0	0	1	2
Excavation (C/D ratio ≥ 0.5)	8	4	1	0	1	0	0	1	1
Coloboma	1	1	0	0	0	0	0	0	0
Hypoplasia	1	0	0	0	0	0	0	0	1
Optic nerve head paleness	8	2	0	2	1	0	1	0	2
Optic atrophy	5	1	0	1	1	0	1	0	1
Retinopathy	1	0	0	1	0	0	0	0	0

*BCVA* best-corrected visual acuity, *C/D ratio* cup to optic disc ratio, *SEA* spherical equivalent of ametropia

**Table 2:** Patients with permanent visual impairment of both eyes (*n* = 7) in the total NS cohort (*n* = 105)

Patient			Vision and refraction			External ocular features				Ocular alignment and motility		Anterior segment	Posterior segment		Genetic findings		
	Gender	Age (years)	Visual acuity (BCVA)		Ametropia (SEA) or astigmatism	Epicanthus	Hypertelorism	Ptosis	Slanting eyelid fissures	Strabismus	Abnormal motility	Abnormality	Optic nerve abnormality	ONHC/D ratio	Mutation	Nucleotide change	Amino acid change
No.	M/F		RE	LE	≥ 1D												
1	F	10	0.3	0.3	Myopia	NR	Yes	Yes	Downward	Yes	SOM palsy RE	No	Atrophy	NR	*RAF1*	*c.1837C > G*	*p.Leu613Val*
2	M	36	0.1	0.1	Myopia	Yes	Yes	Yes	NR	Esotropia	Nystagmus	NR	Atrophy	> 0.5	*SHOC2*	*c.4A > G*	*p.Ser2Gly*
3	M	17	0.3	0.05	Hyperopia	Yes	Yes	Yes	Downward	Esotropia LE	Nystagmus	No	Hypoplasia	NR	No genetic analysis		
4	M	7	0.2	0.16	Hyperopia	NR	NR	Yes	NR	Esotropia	Nystagmus	No	No	NR	*KRAS*	*c.40G > A*	*p.Val14Ile*
5	F	11	0.3	0.3	Hyperopia	NR	Yes	NR	Downward	Exotropia	NR	NR	NR	NR	*SHOC2*	*c.4A > G*	*p.Ser2Gly*
6	M	12	< 0.3	< 0.3	NR	Yes	Yes	Yes	Downward	NR	NR	NR	Atrophy	NR	No mutation identified*		
7	M	19	0.15	0.15	Myopia (> 5D) and astigmatism	NR	NR	NR	NR	Yes	NR	NR	NR	NR	*KRAS*	*c.65A > G*	p.Gln22Arg

Ocular findings were bilateral, if not specified with RE or LE; *BCVA* best-corrected visual acuity, *C/D* cup to disk ratio, *F* female, *LE* left eye, *M* male

*No* feature absent, *NR* feature not recorded or examined, *ONH* optic nerve head, *RE* right eye, *SEA* spherical equivalent of ametropia, *SOM* superior oblique muscle

*PTPN11, *KRAS, BRAF*, *SOS1*, *CBL*, *SHOC2*

Refractive errors, defined as spherical equivalent of ametropia (SEA) of one diopter or more, showed myopia (25 patients), hyperopia (39 patients), and astigmatism (35 patients). High refractive errors, defined as 5 diopters or more, were found for myopia in 5 patients, for hyperopia in 2 patients, and for astigmatism in 3 patients, mainly associated with a causative *PTPN11* mutation.

In 3 patients with a delay in first presentation to an ophthalmologist, ocular abnormalities were found. All 3 of them were diagnosed with Noonan syndrome early in childhood. They were referred to an ophthalmologist for the first time at the ages of 17 to 20 years. One of them was diagnosed with visual impairment due to congenital optic nerve hypoplasia and the other 2 were diagnosed with high corneal astigmatism resulting in keratoconus. In retrospect, visual complaints were noticed earlier in childhood.

External ocular features were frequently found without ophthalmological examination, mostly described by pediatricians and clinical geneticists. The findings included hypertelorism (63 patients), ptosis (55 patients), and downslanting palpebral fissures (39 patients). Other ophthalmological abnormalities included strabismus (40 patients) and nystagmus (16 patients). Anterior segment abnormalities included keratoconus (4 patients), different types and densities of cataract (3 patients), and posterior embryotoxon (2 patients). The posterior ocular segment showed abnormalities of the optic nerve head (ONH), including ONH excavation (8 patients), ONH coloboma (1 patient), ONH hypoplasia (1 patient), and ANH paleness (8 patients) diagnosed as optic nerve atrophy in 5 of them.

In 50 patients, a *PTPN11* mutation was found with genetic testing. These patients were diagnosed with ocular manifestations in the different categories including refractive errors, external ocular features, ocular alignment and motility, and abnormalities in the anterior and posterior ocular segment. No visual impairment was found in the patients with a *PTPN11* mutation. In the NS patients due to a *SOS1* mutation, the most frequent ocular findings were hypertelorism and ptosis. We also found prominent corneal nerves. In the group with NS due to a *RAF1* mutation, we found 1 patient with a unilateral exudative retinopathy (Coats disease).

## Discussion

In 2016, we published our prospective study of ocular manifestations in 25 Noonan syndrome patients [20]. The present retrospective cohort shows a larger group, with more heterogeneity in the patients and more information on the causative molecular findings. This is a retrospective cohort, and therefore we have missing data and there might be a response bias. Nevertheless, it is important to show a larger group of Noonan syndrome patients including their genetic results, and we hypothesized that we could confirm the ocular findings from our prospective cohort.

Visual impairment (binocular BCVA < 0.3) was seen in 7 patients. The cause of the visual impairment is probably a developmental disorder of the optic nerves, presenting with optic nerve atrophy and optic nerve hypoplasia, associated with nystagmus and strabismus. In the prospective study, we had 1 visually impaired patient [20]. This patient had NS due to a *BRAF* mutation. In this cohort, we show visually impaired patients with a *RAF1*, *SHOC2*, or *KRAS* mutation. It is remarkable that in 5 patients with NS (with loose anagen hair) due to a *SHOC2* mutation, 2 patients are visually impaired. In the 4 patients with a KRAS mutation, also 2 patients are visually impaired. The cohort is too small for genotype-phenotype correlations but it shows that visual impairment is found in patients with NS due to a *SHOC2*, *KRAS*, and *RAF1* mutation. In the 50 *PTPN11*-positive patients, no visual impairment is found. In 2 patients with NS due to a *PTPN11* mutation, keratoconus is found and the patient with iris coloboma also has a *PTPN11* mutation. Keratoconus in NS is described in literature in two case reports [2, 7], iris coloboma is described two times [3, 6] and cataract is also described in a few patients with NS [5, 8].

The most frequent external features, are in accordance with the NS cohorts described in literature [1, 8, 10, 12, 20], including hypertelorism, ptosis, strabismus, downslanting palpebral fissures, and epicanthic folds. The external features are important for the clinical diagnosis of NS regarding facial characteristics. These external features/ periorbital findings are mostly diagnosed by pediatricians and clinical geneticists.

Although it is strongly recommended to do a comprehensive ocular examination in all NS patients, still 9 patients never went to an ophthalmologist. In 3 other patients with ocular anomalies and reduced vision, there was a delay of many years before they were referred to an ophthalmologist. In the international NS guidelines [9, 13, 15], referral for an ophthalmological examination after diagnosing NS is recommended for all patients. Examination by an orthoptist and ophthalmologist is also recommended for patients with a suspicion of NS. Repeat the ophthalmological examination in children every 2 years or as advised by the ophthalmologist based on the pathology and the expertise of the ophthalmologist and orthoptist. The high prevalence of ophthalmological anomalies in NS including amblyogenic factors (ptosis, strabismus, astigmatism) makes an early ophthalmological examination necessary [20]. For a successful treatment of amblyopia, it should be diagnosed in time. Some ocular abnormalities in NS patients can occur or increase later in life, for example the keratoconus as described above. The guidelines are published in 2010 and 2013, and that might be an explanation why older NS patients were not referred to an ophthalmological clinic after diagnosing Noonan syndrome. Another reason might be that other symptoms, sometimes life threatening, require more attention early in life (including cardiac abnormalities and feeding difficulties) and therefore vision and other ocular abnormalities do not have priority.

In conclusion, various ocular abnormalities in childhood, including low visual acuity of both eyes leading to visual impairment, are found in a large cohort of patients with Noonan syndrome. Permanent visual impairment is mainly caused by binocular optic nerve abnormalities and nystagmus is found in patients with a mutation in *RAF1*, *SHOC2*, or *KRAS*. Timely screening for ocular abnormalities will help to diagnose NS and facilitates early treatment of potentially vision threatening abnormalities. For children with visual impairment prompt rehabilitation is indispensable for developmental purposes. Therefore, an ophthalmologist should examine all patients suspected of, or diagnosed with NS.

## Abbreviations

BCVA: Best-corrected visual acuity
NS: Noonan syndrome
ONH: Optic nerve head
SEA: Spherical equivalent of ametropia
